# *Kr-h1* Encoding Juvenile Hormone Transcription Factor Impacts Reproductive Functions in *Coccinella septempunctata*

**DOI:** 10.3390/insects17060577

**Published:** 2026-06-01

**Authors:** Ying Cheng, Yuhang Zhou, Cao Li

**Affiliations:** 1 Institute of Plant Protection, Guizhou Provincial Academy of Agricultural Sciences, Guiyang 550006, China; 2 Guizhou Branch of State Key Laboratory for Biology of Plant Diseases and Insect Pests, Guiyang 550006, China; 3 Guizhou Provincial Laboratory of Green Technology and Application Engineering of Plant Protection, Guiyang 550006, China; 4 Guizhou Provincial Pollution-Free Engineering Center of Plant Protection, Guiyang 550006, China

**Keywords:** *Krüppel homolog 1*, *Coccinella septempunctata*, RNAi, juvenile hormone, reproductive function

## Abstract

The gene encoding juvenile hormone transcription factor Krüppel homolog 1 (*Kr-h1*) was studied for its effects on ovary development and reproduction in the ladybug beetle, *Coccinella septempunctata*. *Kr-h1* expression in *C. septempunctata* was evaluated in females supplied with a juvenile hormone (JH) amended diet using quantitative PCR, and the function of *Kr-h1* in female reproduction was assessed using RNAi technology. Expression of *Kr-h1* in females supplied with a JH diet was significantly higher at 5 and 10 d than in females not supplied with JH. At 5 and 10 d post-injection with Kr-h1-dsRNA, *Kr-h1* expression levels were 30.97% and 38.32% lower, respectively, than expression in controls injected with GFP-dsRNA. Development of ovaries and vitellogenesis in ladybugs microinjected with Kr-h1-dsRNA was significantly delayed in comparison to controls. At 20 d post-injection, mean egg production decreased by 28.74% relative to controls. These results prove that *Kr-h1* has a vital role in modulating ladybug fecundity by impacting ovary development and egg production.

## Simple Summary

Juvenile hormone (JH) is a gonadotropin that has roles in metamorphosis, growth, and reproduction. The genes encoding transcription factor Krüppel homolog 1 (*Kr-h1*) and the JH receptor gene (methoprene-tolerant, *Met*) have significant functions in insect reproduction. *Coccinella septempunctata* L. (Coleoptera: Coccinellidae) is an important natural enemy of aphids. RNA interference (RNAi) mediated knockdown of *Met* in *C. septempunctata* females caused a significant downregulation of *Met* expression, delayed ovary development, and reduced fecundity when compared to the control group. However, it remains unclear how *Kr-h1* modulates ovary development and fecundity in ladybugs. In the current study, RNAi was used to evaluate the regulatory role of *Kr-h1* in ladybug reproduction. Our findings provide further insight into how the JH signaling pathway modulates insect reproduction and are relevant to the artificial rearing of this important predator.

## 1. Introduction

Insect-produced juvenile hormone (JH) is secreted by the corpora allata and regulates insect growth, reproduction and metamorphosis [1,2,3,4]. In adult females, JH promotes the synthesis of fat bodies, the secretion of vitellogenin (*Vg*), and facilitates Vg absorption by the ovaries [5,6]. After secretion from the corpora allata, JH binds to carrier proteins and travels through the bloodstream. JH binds to specific receptors such as the Germ cell-expressed (Gce) and Met protein, which leads to the transcription of target genes, including *Kr-h1*; ultimately, this interaction impacts insect growth and development, morphological changes, and reproduction [7,8]. Current research has found that the *Kr-h1* is necessary for maintaining nymphal characters in *Riptortus pedestris* [9] and *Acyrthosiphon* pisum [10]. Knockdown of *Kr-h1* at the larval instar stages led to deformed prepupae and misshapen pupae [11]. In *Liposcelis entomophila*, silencing *Met* and *Kr-h1* remarkably reduced the transcription of *LeVg* and *LeVgR*, disrupted the production of Vg in fat body and the uptake of Vg by oocytes, and ultimately led to a decline in fecundity [12]. JH acts through the Met-Kr-h1 signaling pathway operating in antennal lobes to promote pheromone information processing and consequently the display of sexual behavior in synchronization with fertility to optimize male reproductive fitness [13]. In addition to its role in regulating insect growth, development and reproduction, *Kr-h1* impacted foraging behavior and nerve cell formation in *Apis mellifera* and *Drosophila* [14,15].

In the insects *Locusta migratoria* and *Colaphellus bowringi*, the application of JH analogs promoted *Met* and *Kr-h1* transcription, thereby facilitating the expression of *Vg* and its receptor gene, *VgR*, which influenced ovary development and maturation [16,17]. In *Agrotis ipsilon*, the injection of JH inhibitors and analogs confirmed that *Kr-h1* was regulated by JH and influenced sexual behavior [18].

The seven-spot ladybug, *C. septempunctata*, is a dominant natural enemy of important pests including aphids, whiteflies, leafhoppers, and spider mites. When *C. septempunctata* females were treated with the JH analog ZR-512, oocyte growth and Vg content in the hemolymph increased significantly [19]. We previously reported that supplementation of an artificial diet with JH increased egg production by fourfold as compared to an artificial diet lacking JH [20]. RNA interference (RNAi) technology leverages double-stranded RNA (dsRNA) to induce sequence-specific gene silencing, enabling precise targeting of essential genes in insects [21]. RNAi-mediated knockdown of *Met* in *C. septempunctata* females caused a significant downregulation of *Met* expression, delayed ovary development, and reduced fecundity when compared to the control group [22]. However, it remains unclear how *Kr-h1* modulates ovary development and fecundity in ladybugs. In the current study, RNAi was used to evaluate the regulatory role of *Kr-h1* in ladybug reproduction. Our findings provide further insight into how the JH signaling pathway modulates insect reproduction and are relevant to the artificial rearing of this important predator. At the same time, it will provide a theoretical basis for the development of new RNAi drugs targeting the reproduction of pests [21].

## 2. Materials and Methods

### 2.1. Insects

Ladybugs were collected from the wild wheat fields and raised for more than 20 generations in the laboratory of the Guizhou Provincial Institute of Plant Protection. Seedlings of broad bean, *Vicia faba*, were cultivated in plastic boxes and used to rear the host aphid, *Aphis craccivora*. Ladybug larvae were transferred to the boxes and reared until pupation. The conditions for rearing were 25 °C, 60–70% RH, and a 14:10 h light: dark photoperiod. Newly emerged 1 d-old ladybugs were selected and reared separately on aphids or supplied with artificial diets.

### 2.2. Artificial Diets

Diet A has been described previously [20] and consisted of the following: 15 g powdered milk, 105 g pig liver, 2 g olive oil, 45 g sucrose, 10 g egg, 7.5 g powdered yeast, 2 g corn oil, 0.5 g cholesterol, 5 g casein, 4.5 g powered protein, 1 g each of vitamin E and C, 7.5 g honey, 6.2 g agar, 370 mL purified water, and 1.0 g sorbic acid (99.0% active content).

Diet B consisted of diet A supplemented with 3 μL of 65% juvenile hormone III (Sigma-Aldrich, Co., St. Louis, MO, USA).

Diet preparation involved homogenizing fresh pig liver into a paste with a JJ-2 tissue homogenizer and adding the remaining ingredients to a 1 L beaker with the exception of water, agar and sorbic acid. Agar was weighed, transferred to a flask containing the purified water, heated until dissolved, and then supplemented with sorbic acid. After thoroughly mixing at 40–50 °C, the agar solution was quickly poured into the 1 L beaker containing the other ingredients. The mixture was rapidly stirred, dispensed into Petri dishes and cooled to ambient temperature; this resulted in a semi-solid artificial diet that was stored at 4 °C.

### 2.3. Expression Profile of Kr-h1 in Adult Female Ladybugs

Female adults were reared on aphids or diets A or B, collected at 5 and 10 d, frozen in liquid nitrogen and stored at −80 °C. Individual samples comprised four adult females, and samples were replicated three times. RNA was isolated from samples with the Eastep^®^ Super Total RNA Extraction Kit as instructed by the manufacturer (Promega, Beijing, China). The Bio-Rad iScript cDNA Synthesis Kit (Bio-Rad, Hercules, CA, USA) was utilized to produce template cDNA. Primer sets were sourced from Sangon Biotech Co., Ltd. (Shanghai, China). The Kr-h1-F/Kr-h1-R primer set (Table 1) was designed based on the cloned *Kr-h1* sequence, which is accession no. OR183710 in the National Center for Biotechnology Information (NCBI) database. *Actin* primers were synthesized as described previously [5]. The qPCR reaction was executed in a 20 μL volume containing the following: forward and reverse primers, 2 μL of each; cDNA template, 2 μL; Sso Advanced Universal SYBR Green Supermix (Bio-Rad, Hercules, CA, USA), 10 μL; and double-distilled water, 4 μL. PCR was executed as follows: 3 min at 95 °C; 40 cycles at 95 °C for 5 s; and primer annealing temperatures for 30 s. Melting curves were generated to verify primer specificity. Each biological replicate included three technical replicates. Transcription levels of *Kr-h1* were normalized using the 2^−∆∆Ct^ method [23].

### 2.4. RNAi Methodology

#### 2.4.1. Synthesis of Kr-h1-dsRNA

Primers Kr-h1-dsRNA-F and Kr-h1-dsRNA-R were designed within the functional region of *Kr-h1* (NCBI accession no. OR183710) using Primer 5.0 software. T7 promoters were added to the 5′ ends of primers (Table 1). Specific primers for *GFP* encoding green fluorescent protein are shown in Table 1. Specific fragments of *Kr-h1* and *GFP* were amplified from *C. septempunctata* cDNA as directed in the Phanta^®^ Max Super-Fidelity DNA Polymerase Kit (Vazyme, Nanjing, China). Amplicons containing *GFP* and *Kr-h1* were separated by agarose gel electrophoresis and purified using the Vazyme FastPure^®^ Gel DNA Extraction Mini Kit. The Transcript Aid T7 High Yield Transcription Kit (Thermo Fisher Scientific, Lenexa, KS, USA) was used to synthesize GFP-dsRNA and Kr-h1-dsRNA. Reactions were conducted in 40 μL containing the following: DEPC-treated water, 6 μL; 5× Transcript Aid Reaction buffer, 8 μL; template DNA, 6 μL; Transcript Aid Enzyme mix, 4 μL; and ATP/CTP/GTP/UTP mix, 16 μL. Reactions were incubated at 37 °C for 4 h, treated with DNase I, and incubated for an additional 15 min. The reaction was terminated by adding two microliters of 0.5 M EDTA (pH 8.0); this was followed by a 10 min incubation at 65 °C. The synthesized dsRNA was purified, recovered, and stored at −80 °C.

#### 2.4.2. dsRNA Injection

Newly emerged, 1 d-old *C. septempunctata* female adults were injected with the Eppendorf Transferman 4R microinjection system (Eppendorf, Hamburg, Germany). Female ladybugs were transferred to a 250 mL container, anesthetized with CO_2_ gas, and injected with one microliter (4500 ng/µL) of Kr-h1-dsRNA or GFP-dsRNA at the intersegmental membrane between the third and fourth abdominal segments. After injection, the needle was held in place for 5 s to minimize leakage. Insects injected with GFP-dsRNA were considered controls. Each treatment comprised 50 females, and each experiment was replicated three times.

#### 2.4.3. Effects of RNAi on Female Adults

After dsRNA injection, ladybugs were reared on aphids. Adult females were collected at 5 and 10 d post-injection, and RNA extraction, cDNA synthesis, and qPCR were executed as outlined in Section 2.3. Each sample consisted of three biological replicates, with four insects per replicate. Female adults collected at 5 and 10 d post-injection were dissected in PBS buffer, ovaries were removed, and samples were analyzed by stereomicroscopy. Image View was utilized to determine ovary lengths and dimensions of left and right ovarioles. The length of the ovary was measured from the top of the ovary to the bottom of the egg chamber, and the width of the ovary was the sum of the widths of the left and right egg chambers. Thirty female adults were dissected per treatment at each time point.

To measure fecundity, injected females were paired with males with 10 pairs/treatment and three replicates for a total of 30 pairs. The quantity of eggs laid over a 20 d post-injection period was recorded.

### 2.5. Data Analysis

Datapoints were analyzed using DPS 18.0 [24] with ANOVA (one-way analysis of variance). The LSD (Least Significant Difference) method was utilized to assess significance.

## 3. Results

### 3.1. JH Affects Kr-h1 Expression in C. septempunctata

**Figure 1:**
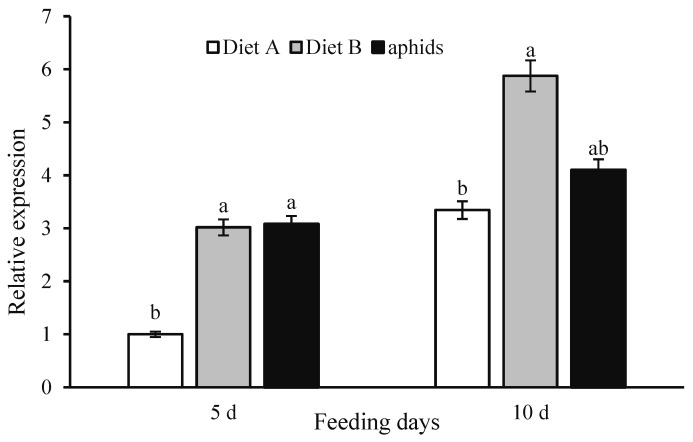
Transcript levels of *Kr-h1* in ladybug females at 5 and 10 d after consumption of diets A, B, and aphids. Diet B consisted of ingredients in diet A plus the addition of JH. Datapoints represent means ± SD, and bars with different letters represent significance at *p* < 0.05 after analysis with the LSD test.

*Kr-h1* expression was significantly elevated in ladybug females reared on diet B containing JH as compared to insects feeding on diet A without JH (Figure 1). After a 5 and 10 d feeding period, *Kr-h1* transcript levels in the JH-supplemented group (diet B) were 3.02- and 1.76-fold higher than expression in ladybugs supplied with diet A, which lacked JH. At 5 d, *Kr-h1* transcript levels in ladybugs feeding on diet B were not significantly different from expression in insects reared on aphids. At 10 d, *Kr-h1* expression in the JH-supplemented group was greater than levels in ladybugs feeding on aphids, but this difference was not significant.

### 3.2. Kr-h1-dsRNA Affects Kr-h1 Expression in C. septempunctata

**Figure 2:**
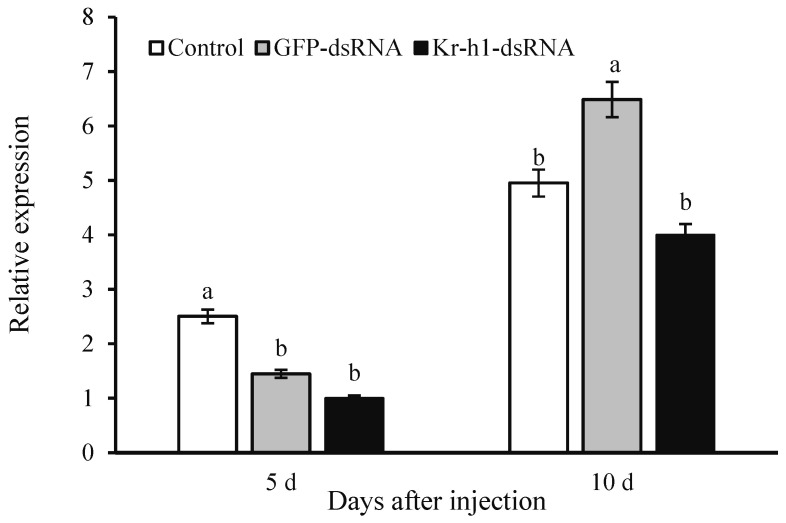
*Kr-h1* expression in ladybug females at five and ten days following injection with Kr-h1-dsRNA and GFP-dsRNA. Datapoints represent means ± SD, and bars with different letters represent significance at *p* < 0.05. Controls consisted of non-injected females.

*Kr-h1* transcript levels in Kr-h1-dsRNA-injected females were 30.97% and 38.32% lower than expression in the GFP-dsRNA-treated control group at 5 and 10 d after injection, respectively (Figure 2). *Kr-h1* transcript levels in the group injected with Kr-h1-dsRNA were 60.07% lower than the control group at day 5 but increased by 19.25% at day 10 when compared to the non-injected control group.

### 3.3. Impact of Kr-h1-dsRNA Injection on Ovary Development

**Figure 3:**
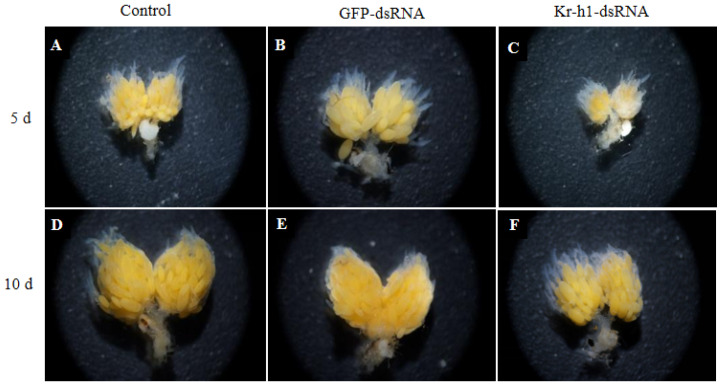
Development of ovaries in ladybug females after injection with GFP-dsRNA or Kr-h1-dsRNA. Panels (**A**–**C**) depict ovaries in females at 5 d, and (**D**–**F**) show ovaries at 10 d after microinjection with Kr-h1-dsRNA and GFP-dsRNA. The control group shows ovary development in noninjected females.

**Figure 4:**
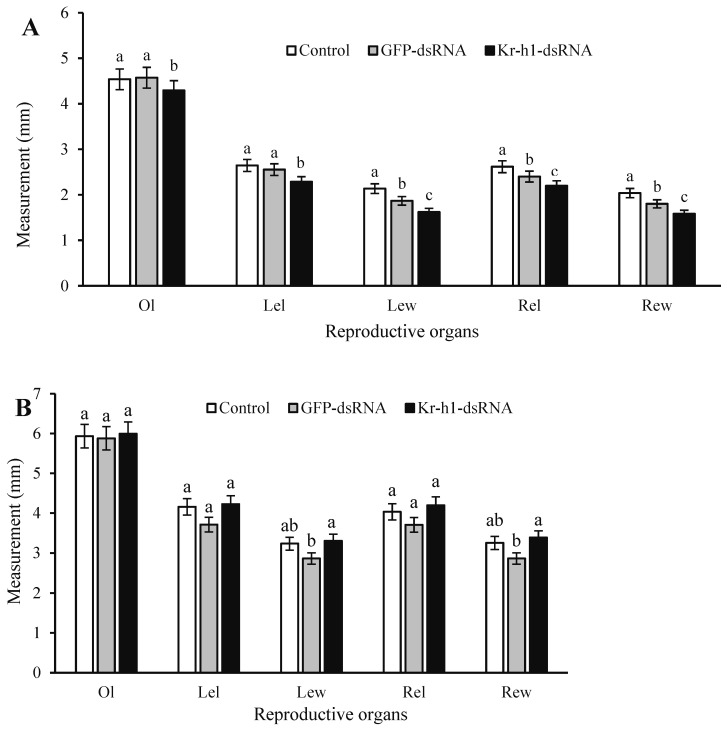
Development of ovaries in ladybug females injected with Kr-h1-dsRNA or GFP-dsRNA. (**A**) Ovary measurements at 5 d and (**B**) 10 d after microinjection. Abbreviations: Length of ovaries, OL; length of the left egg chamber, Lel; width of the left egg chamber, Lew; length of the right egg chamber, Rel; and width of the right egg chamber, Rew. Datapoints show means ± SD, and bars topped with different letters indicate a significant difference at *p* < 0.05.

Dissected ovaries examined at five and ten days following injection with Kr-h1-dsRNA exhibited delayed development relative to the GFP-dsRNA-treated group (Figure 3). The ovaries of the GFP-dsRNA-treated group and controls contained numerous mature eggs, while the Kr-h1-dsRNA-treated group had fewer mature eggs and ovarioles with a deflated appearance. Measurements at day 5 post-injection (Figure 4A) indicated that the Kr-h1-dsRNA-injected group exhibited decreases in the length of ovaries (OL), length of the left chamber (Lel), width of the left egg chamber (Lew), length of the right egg chamber (Rel), and width of the right egg chamber (Rew) by 6.11%, 10.55%, 13.24%, 8.44%, and 12.21% as compared to the GFP-dsRNA-injected group, respectively, there were significant difference (*p* < 0.05). However, at day 10 post-injection (Figure 4B), OL, Lel, Lew, Rel and Rew in the Kr-h1-dsRNA-injected group increased by 1.95%, 13.81%, 15.59%, 13.28%, and 18.44% when compared to the GFP-dsRNA-treated group, respectively, and significant differences were observed for the left and right egg-chamber width (*p* < 0.05).

### 3.4. Kr-h1-dsRNA Impacts on C. septempunctata Fecundity

**Figure 5:**
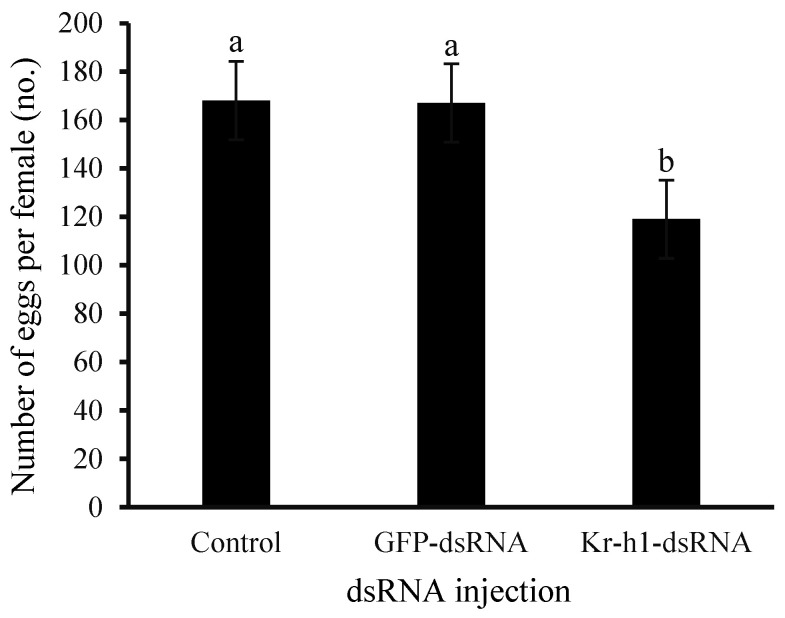
Ladybug fecundity 20 d after microinjection with Kr-h1-dsRNA or GFP-dsRNA. The control represents noninjected females. Datapoints show means ± SD, and columns with different letters show significant differences at *p* < 0.05.

The average number of eggs laid by each female over a 20 d sampling period was 119 in Kr-h1-dsRNA-injected females as compared to 167 and 168 eggs per female in the non-injected control and GFP-dsRNA-treated groups, respectively (Figure 5). Statistical analysis indicated that fecundity was significantly reduced in the Kr-h1-dsRNA-injected group (*p* < 0.05), whereas injection with GFP-dsRNA did not impact fecundity.

## 4. Discussion

*C. septempunctata Kr-hl* encoded a 1338 bp open reading frame (ORF) consisting of 445 predicted amino acids, which showed high similarity to orthologs in other insect species and contained eight highly conserved Zn-finger motifs for DNA-binding. Phylogenetic analysis indicated that *C. septempunctata Kr-hl* and *Aethina tumida Kr-hl* clustered together in one branch [25].

Prior reports have documented that *Kr-h1* functions in mediating JH signaling, thereby regulating metamorphosis, reproduction, and other physiological functions in insects. For example, treatment of fruit flies and silkworms with JH analogs promoted *Kr-h1* expression, which led to delayed larval pupation [26,27]. *Kr-h1* expression in *Bactrocera dorsalis* responded to JH analogs in the late larval and pupal stages, and inhibiting JH synthesis or expression levels resulted in decreased *Kr-h1* expression and premature larval-to-pupal transitions [28]. In the brown planthopper, *Kr-h1* was actively transcribed in brains, intestines, wings, and ovaries, suggesting a crucial role in neural pathways and reproductive development [7]. In our previous study, high expression levels were observed for *Kr-h1* in ladybug female adults at 1 d after emergence, followed by decreased expression at 5, 10, 15, and 20 d [25]. Interestingly, *Kr-h1* expression levels increased again at 25 and 30 d and were 2.27- and 3.82-fold higher than levels on day 1, respectively. *Kr-h1* expression was higher in ladybug heads than in the thorax, fat bodies, and ovaries, further indicating a potential role for *Kr-h1* in neural pathways [25]. In the present study, supplementation of artificial diets with JH resulted in *Kr-h1* expression levels that were 3.02- and 1.76-fold higher on days 5 and 10, respectively, than expression in females fed on an artificial diet lacking JH. Thus, our data indicate that supplementation of artificial diets with JH promoted *Kr-h1* transcription in *C. septempunctata*.

Many reports describe the regulatory functions of *Kr-h1* and *Met* on vitellogenesis, ovary development, and fecundity by utilizing RNAi to silence these genes. Studies employing RNAi have shown that the knockdown of *Kr-h1* or *Met* in moths [29], planthoppers [30], and locusts [31] resulted in decreased *Vg* and *VgR* expression, delayed ovary development, and significantly reduced egg production. Furthermore, microinjection of Kr-h1-dsRNA into 3rd instar nymphs of the brown planthopper resulted in malformed wings and deformed genitalia in both adult females and males [32]. Yao et al. [33] confirmed this finding by showing that *Kr-h1* knockdown in 4th instar nymphs of the brown planthopper resulted in genital deformities in females. Previously, we showed that *Met* knockdown in ladybug females decreased the development of ovaries and significantly reduced fecundity [22]. In this study, expression levels of *Kr-h1* in *C. septempunctata* males were reduced 5 d after injection with Kr-h1-dsRNA, and expression was significantly lower than that of the control group. At 10 d post-injection, *Kr-h1* expression remained lower than the control group, but the difference was not statistically significant. It remains possible that the injected Kr-h1-dsRNA was enzymatically cleaved into siRNA; also, cellular nucleases may have progressively degraded Kr-h1-dsRNA, leading to a decrease in RNA interference efficiency and a gradual recovery of *Kr-h1* expression. At five days after microinjection with Kr-h1-dsRNA, ovary development was delayed and there were fewer mature eggs than present in GFP-dsRNA-injected females. At 10 days after injection with Kr-h1-dsRNA, an increased number of empty ovarioles was observed, and ovary lengths and widths were greater than those in the GFP-dsRNA-injected group. Females injected with Kr-h1-dsRNA could still mate and lay eggs, but fecundity was reduced. Given the relatively long pre-oviposition and oviposition periods in *C. septempunctata*, the interference efficiency of Kr-h1-dsRNA may gradually decrease over time. Additionally, compared to *Met* knockdown [22], ovary development in *C. septempunctata* was less impacted by *Kr-h1* knockdown; whether *Met* played a dominant role in the reproductive regulation of the ladybugs still needs to be further verified by injecting several different concentrations and different dsRNA sequences.

In this study, RNAi was performed through the injection of dsRNA. The injection of dsRNA involves trauma, inconvenient operation, and strict storage requirements. However, oral RNAi is much more convenient to perform, but dsRNA is easily degraded by gastrointestinal nucleases, resulting in low bioavailability. Recently, various nanoparticles in oral RNAi in insects have been explored [34]. Qiao et al. [35] designed a nanoparticle chitosan-polyethylene glycol-carboxyl (CS-PEG-COOH), which can spontaneously assemble with dsRNA to form the dsRNA/CS-PEG-COOH complex. CS-PEG-COOH was able to prevent dsRNA from being degraded by midgut fluid or RNase A, thereby significantly improving the dsRNA stability under various environmental conditions. The co-delivery system of nanocarriers and dsRNA is a promising approach for advancing insect RNAi.

In this study, the regulatory role of *Kr-h1* in ladybug reproduction was confirmed using RNAi technology. This finding will facilitate future studies aimed at elucidating other regulatory mechanisms that the JH signaling pathway uses to modulate insect reproduction. Furthermore, our results provide insight on the complex relationship among JH titers and *Kr-h1* transcription levels in *C. septempunctata*, which could be utilized to further improve artificial diets for this important predator.

## Figures and Tables

**Table 1:** Primers utilized used in this study.

Primer Name	Primer Sequence (5′-3′)	Annealing Temperature
Kr-h1-F	AACCTTTCGAGTGCCCTGAAT	58.3
Kr-h1-R	ATGCCTCCTCCTGAACCTACT	
Kr-h1-dsRNA-F	taatacgactcactatagggAAGGATCTCACCACCGACAC	
Kr-h1-dsRNA-R	taatacgactcactatagggGGCTCCGTTTGTTCTGGTAA	
GFP-dsRNA-F	taatacgactcactatagggGCCAACACTTGTCACTACTT	
GFP-dsRNA-R	taatacgactcactatagggGGAGTATTTTGTTGATAATGGTCTG	
Actin-F	GATTCGCCATCCAGGACATCTC	60.0
Actin-R	TCCTTGCTCAGCTTGTTGTAGTC	

Lowercase letters represent the T7 promoter sequence.

## Data Availability

The original contributions presented in this study are included in the article. Further inquiries can be directed to the corresponding author.
